# Onset of Guillain Barré Syndrome in a Pregnant Woman: A Report of a Rare Occurrence

**DOI:** 10.7759/cureus.77715

**Published:** 2025-01-20

**Authors:** Victoria K Corder, Briana S Collins, Travis Smith

**Affiliations:** 1 Obstetrics and Gynecology, Lake Erie College of Osteopathic Medicine, Bradenton, USA; 2 Clinical Curriculum Integration and Assessment, Lake Erie College of Osteopathic Medicine, Bradenton, USA

**Keywords:** acute inflammatory demyelinating polyradiculoneuropathy, acute transverse myelitis (atm), demyelinating disorders, guillain- barré syndrome, paresthesia in pregnancy

## Abstract

Guillain Barré Syndrome (GBS) is a rare neurological disorder in which the body’s immune system abnormally attacks the peripheral nervous system. When this occurs, signal transduction between the central nervous system to the peripheral nervous system is disrupted. Symptoms of GBS can vary, and the exact cause is uncertain. We report a case of a pregnant 24-year-old woman in her third trimester, who presented with paresthesia and weakness that progressed through her postpartum period. Initially misdiagnosed, the case underscores the diagnostic challenges of GBS and highlights the importance of timely intervention. Standard therapies include intravenous immunoglobulin (IVIG) and plasmapheresis; in this instance, our patient received the latter. Despite the delayed diagnosis, she received appropriate treatment and showed improvement. This case highlights the complexity behind diagnosing GBS, particularly in the obstetric population, and the critical need for early recognition and management to improve patient outcomes.

## Introduction

Guillain Barré syndrome (GBS) describes a spectrum of acute immune-mediated polyneuropathies (AIDPs) that cause symmetric muscle paralysis and weakness. There are various subtypes of GBS that are dependent upon the following: type of nerves involved (motor, sensory, autonomic, or cranial) and the extent of nerve fiber damage (axon vs myelin sheath) [1]. The most common form of GBS is acute inflammatory demyelinating polyneuropathy, which involves the destruction of the myelin sheath with symptoms of ascending muscle weakness. Other common forms include acute motor axonal neuropathy (AMAN) and acute motor-sensory axonal neuropathy (AMSAN). The incidence of GBS ranges from 0.4 to 1.7 per 100,000 people annually [2]. For GBS in pregnant patients, it is estimated that it occurs in only 1.2-1.9 per 100,000 people with increased risk in the third trimester and first two weeks of the postpartum period. This elevated risk is due to an increase in a delayed type of hypersensitivity [3].

Symptoms of GBS can vary depending on the specific subtype of the condition. Weakness is one of the most common symptoms and typically occurs in an ascending pattern, starting at the feet and slowly progressing upwards. Initially, individuals may have trouble walking and balancing. By the third week after onset, 90% of people are at their weakest [4]. Depending on the extent of nerve damage, patients can also experience sensory changes, such as paresthesia and heightened sensations of pain. These symptoms, including unexplained bouts of numbness and tingling, often occur early and may lead to delayed diagnosis. Other symptoms may include difficulty with eye movements and vision, pins and needles sensations that occur in the hands and feet, severe pain during the night, or even difficulty swallowing and chewing. Thus, GBS can present with a myriad of symptoms and can subsequently be misdiagnosed until the symptoms progress. We present the case of a pregnant woman who was diagnosed with GBS after an initial misdiagnosis, attributed to her nonspecific and vague presentation.

## Case presentation

A previously healthy 24-year-old female patient, with a past medical history of anemia, G2P1001 at 39 weeks and two days, presented to the emergency department (ED) for a ground-level fall and subsequent injury to her left ankle at home. She described her ankle rolling underneath her but denied hitting her abdomen in the fall. The patient explained that she had been using a walker at home to assist her during her pregnancy due to generalized weakness and intermittent paresthesia in her lower extremities. These symptoms have been occurring for two months, leading to multiple falls. The patient thought that her condition was due to sciatica based on some prior ED visits; however, while she admitted to more classical symptoms such as lower back pain, she denied any focal radiation down either of her lower extremities. In addition to this visit, the patient had also presented to the ED at 36 weeks and two days for a fall at home due to loss of balance and weakness, along with another visit when she was at 35 weeks, with a chief complaint of generalized weakness and dizziness at home. She had a past medical history of iron deficiency anemia but had been missing her iron transfusions since before her ED visit at 35 weeks, due to problems with insurance. Thus, the patient and her family assumed her symptoms correlated with her iron deficiency. She had no other significant medical history, comorbidities, recent illnesses, or contributory family history. She denied use of alcohol, tobacco, and illicit drug use.

The patient was diagnosed with an ankle sprain, provided with a walking boot for stabilization, and recommended to follow up with an orthopedist if symptoms worsened. She was subsequently admitted to labor and delivery one day following the last ED visit, where she requested to be induced six days prior to her anticipated due date. Her vitals at initial presentation to labor and delivery are shown in Table 1, complete blood count (CBC) is shown in Table 2, and fetal heart tracing is shown in Table 3.

**Table 1:** Vitals SpO2, peripheral oxygen saturation

Component	Values
Blood Pressure	126/81 mmHg
Location	Left arm
Pulse	100 bpm
Respirations	18 breaths/minute
Temperature	98.5° F (36.9° C)
SpO2	100%

**Table 2:** Complete blood count WBC, white blood cell count; RBC, red blood cell count; MCV, mean corpuscular volume; MCH, mean corpuscular hemoglobin; MCHC, mean corpuscular hemoglobin concentration; RDW, red cell distribution width; ABO, antigen blood O group system; Rh-, rhesus factor negative

Component	Patient value	Reference range
WBC	6.01	4.80-10.80 10^3/uL
RBC	4.32	4.20 - 5.40 10^6/uL
Hemoglobin	11.3	12.0 - 16.0 g/dL
Hematocrit	35.9	37.0 - 47%
MCV	83.1	80.0 - 99.0 fL
MCH	26.2	27.0 - 33.0 pg
MCHC	31.5	33.0 - 36.5 g/dL
RDW	21.9	11.9 - 17.7%
Platelet Count	193	150 - 400 10^3/uL
ABO	Rh- / O Positive	—

**Table 3:** Fetal heart tracing

Component	Value
Fetal Heart Rate Baseline	135 bpm
Variability	Moderate
Accelerations	Present
Decelerations	Absent
Category of Tracing	1

On the day of admission to Labor and Delivery (L&D), the patient was given a dinoprostone (Cervidil) vaginal insert 10 mg for induction of labor. On day two of her L&D stay, she received a spinal epidural, with no reported complications. Three to four hours after the start of the epidural, the patient had a spontaneous vaginal delivery. She was reported to have lost 100 mL of blood during the labor, with no other complications of note. Her newborn male child received APGAR scores of 9 at one minute and 9 at five minutes.

On postpartum day one, the patient complained of numbness and weakness in her lower extremities, with notable difficulty upon standing and ambulating, per nursing staff. During rounds with Obstetrics and Gynaecology (OBGYN), the patient reported experiencing weakness, along with a “pins and needles” sensation in her feet bilaterally. The patient was referred for occupational therapy (OT) and physical therapy (PT) for evaluation and strength training. PT observed improvement in the patient's strength compared to her initial complaints of weakness; however, she continued to experience bilateral lower extremity weakness and sensory deficits.

On postpartum day two, the patient was unable to walk, with associated weakness, tingling, and a “cold feeling” in her lower extremities bilaterally. She also started to complain of bilateral weakness in her hands, which she revealed had been going on intermittently for about two months. When discussed previously with a midwife, she was provided with a diagnosis of carpal tunnel syndrome. At this point, OBGYN requested a neurology and internal medicine consult. The neurologist’s physical exam found the following (strength scale 0-5, 0 = no muscle contraction identified, 1 = muscle contraction is identified but fails to produce full motion against gravity, 2 = muscle can move the joint in full range of motion in the elimination of gravity, 3 = muscle can move the joint in full range of motion against gravity, 4 = muscle can move the joint in full range of motion with moderate resistance, 5 = muscle can move joint in full range of motion will full resistance): cranial nerves I-XII were intact; upper extremity strength intact both proximally and distally, bilateral lower extremity motor: iliopsoas 2 out of 5, hamstrings 4 out of 5, quadriceps 4 out of 5, dorsiflexion 4 out of 5; lower extremity deep tendon reflexes were absent; plantar flexion bilaterally; cerebellar: finger to nose is without dysmetria, sensation: vibration and pinprick intact, proprioception diminished in left lower extremity. Based on her history and these findings, an MRI of the brain, cervical spine, and thoracic spine with and without IV contrast was ordered.

On postpartum day three, OT stated that the patient began to experience declines in standing balance, functional mobility, and transfer, along with persistent numbness, tingling, and weakness in the lower extremities. PT noted that while the patient was able to ambulate 50 feet compared to her prior 20 feet on postpartum day one, she began to show signs of Trendelenburg (weakness in the gluteus medius and minimus muscles that cause contralateral pelvic drooping while walking [5]), foot drop, and knee locking. A comprehensive metabolic panel (CMP) was ordered, and the results are shown in Table 4.

**Table 4:** Comprehensive metabolic panel BUN, blood urea nitrogen; AST, aspartate aminotransferase; ALT, alanine aminotransferase; eGFR, estimated glomerular filtration rate

Component	Patient value	Reference range
Sodium	137	135-145 mmol/L
Potassium	3.9	3.3-4.9 mmol/L
Chloride	103	101-111 mmol/L
Carbon Dioxide	23.0	21.0-31.0 mmol/L
Anion Gap	11	6-16 mmol/L
BUN	11.0	6.0-25.0 mg/dL
Creatinine	0.36	0.44-1.03 mg/dL
BUN/Creatinine Ratio	30.6	—
Glucose	130	68-99 mg/dL
Calcium	9.8	8.5-10.5 mg/dL
AST	14	10-42 U/L
ALT	13	10-60 U/L
Alkaline Phosphatase	144	42-121 U/L
Protein, Total	7.0	6.7-8.2 g/dL
Albumin	3.50	3.40-5.50 g/dL
Bilirubin, Total	0.40	0.20-1.20 mg/dL
eGFR	145.6	≥60.0 mL/min/{1.73_m2}

**Figure 1:**
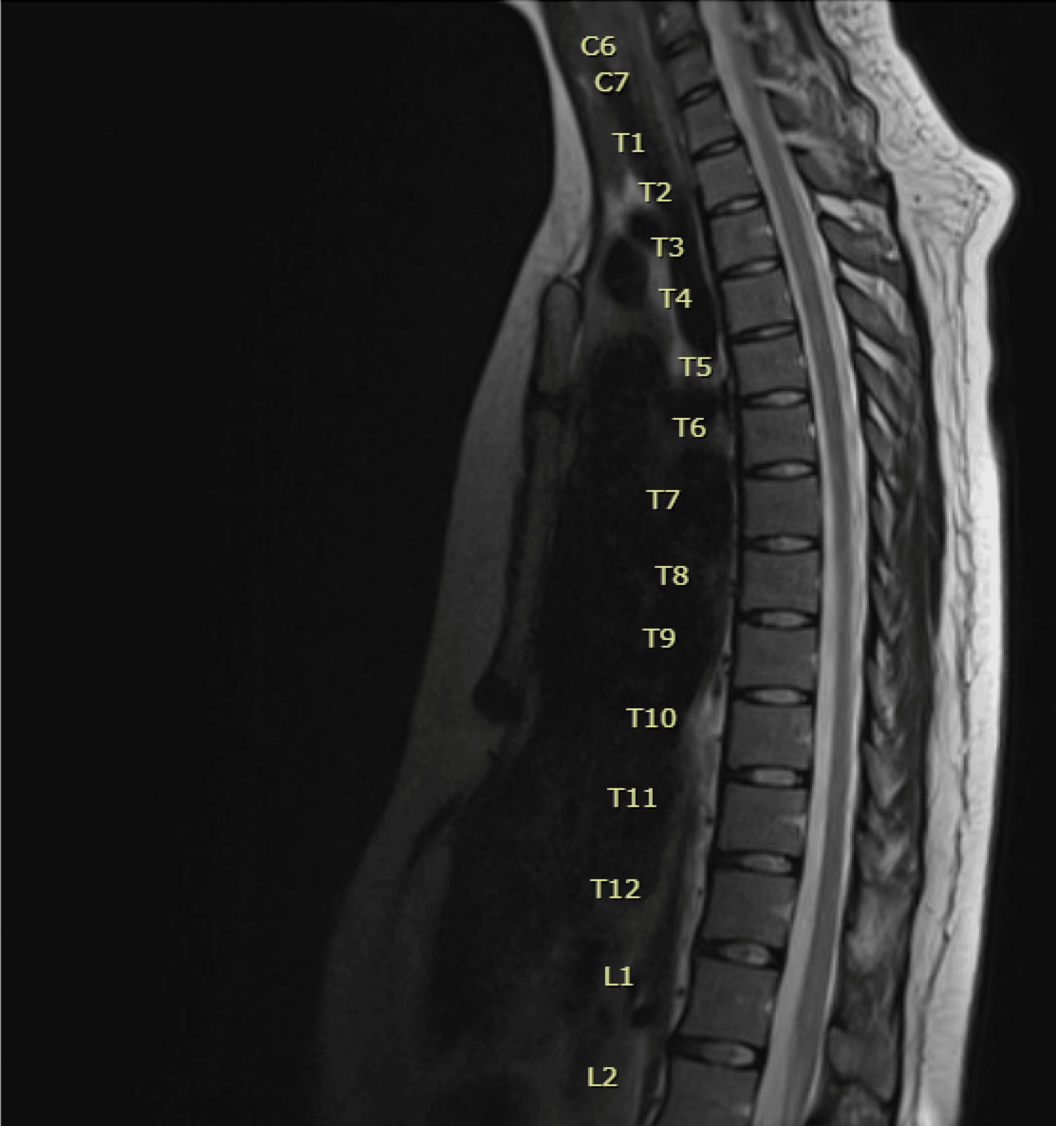
MRI of thoracic spine

**Figure 2:**
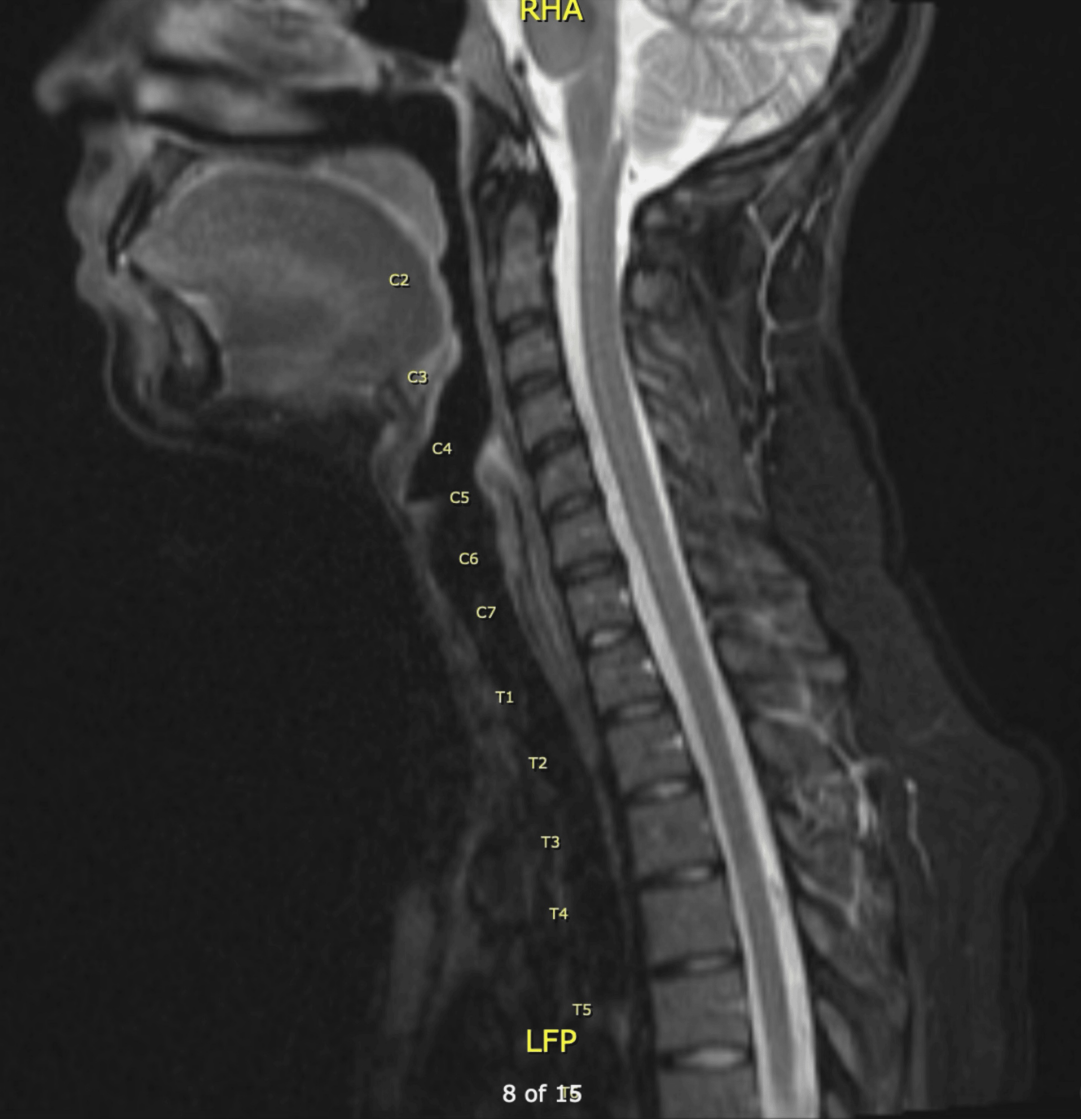
MRI of cervical spine

**Figure 3:**
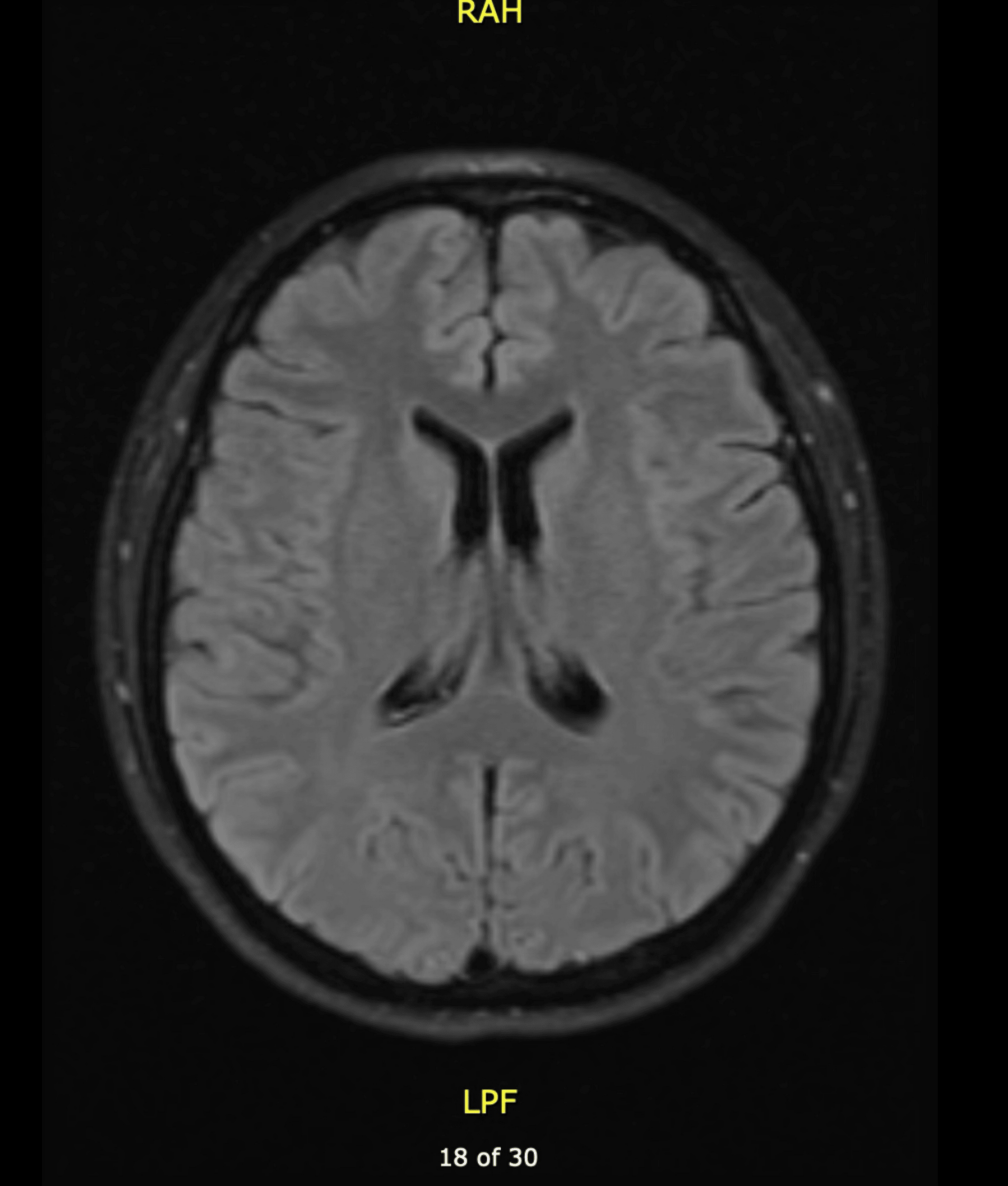
MRI of brain

MRI results were finalized on postpartum day three. Thoracic spine scan with and without IV contrast showed subtle T2 signal change suggesting edema involving the lower half of the thoracic cord, particularly at T12 and L1 suggesting a differential diagnosis of transverse myelitis (Figure 1). The cervical spine scan with and without IV contrast (Figure 2) and brain scan with and without IV contrast MRIs (Figure 3) were reported as normal.

**Figure 4:**
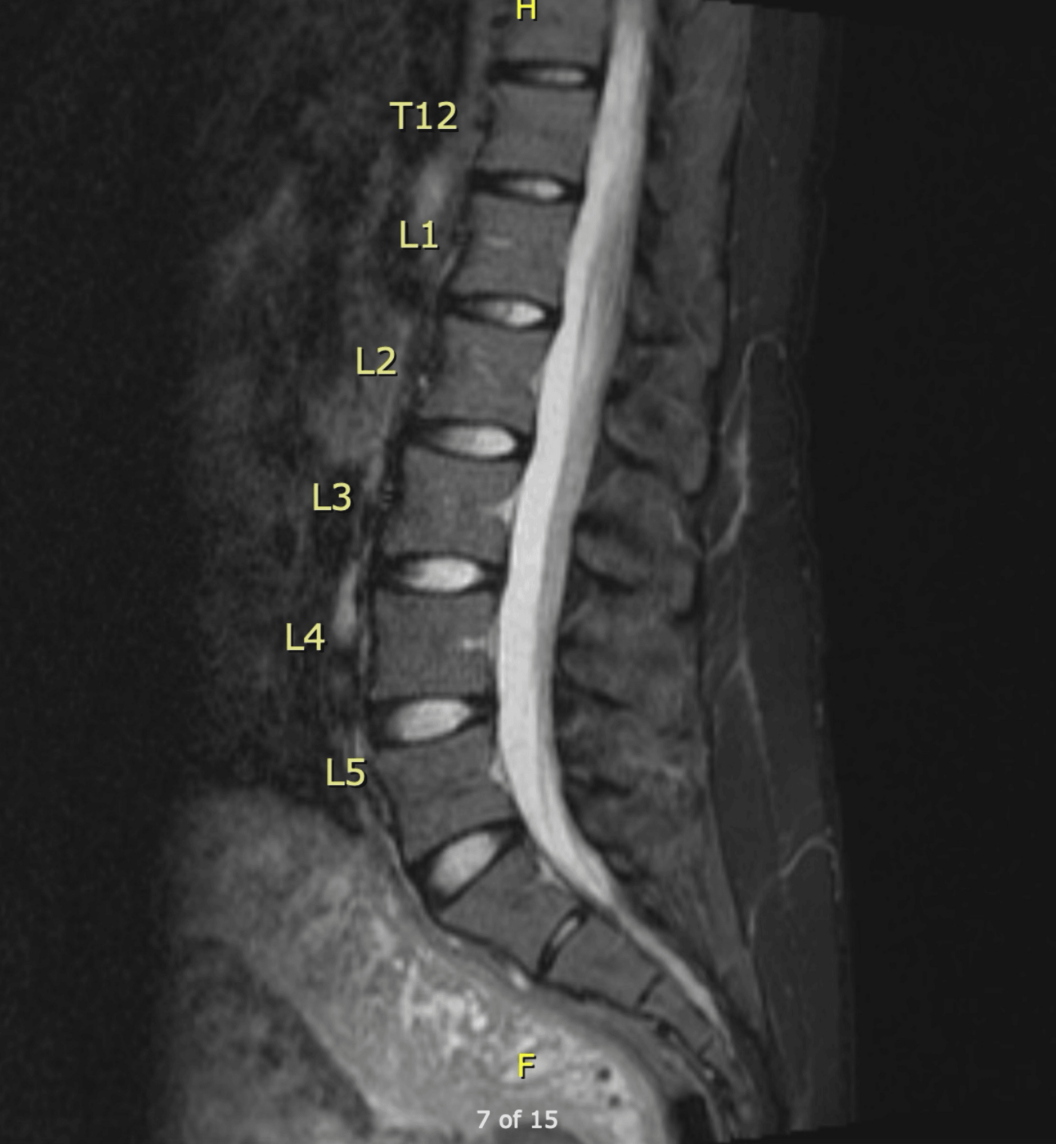
Lumbar spine

Following the MRI results on postpartum day three, a lumbar puncture (CSF results listed in Table 5 and Table 6) and an MRI scan of the lumbar region with and without IV contrast (Figure 4) were ordered on postpartum day four. The lumbar region MRI showed no discrete signal abnormality or pathologic enhancement of the conus and cauda equina. The subtle T2 signal change involving the conus seen on the recent thoracic spine MRI was not identified on the lumbar region MRI and may have been related to motion artifact. Based on the lumbar region MRI findings, the elevated protein in the CSF, and the patient's clinical exam and history, a diagnosis of GBS was made.

**Table 5:** CSF analysis CSF, cerebral spinal fluid; WBC, white blood cell count; IgG, immunoglobulin G; AQP4, aquaporin-4

COMPONENT	VALUE	REFERENCE RANGE
Protein, CSF	>600.00	15.0 - 45.0 mg/dL
WBC, CSF	2	0 - 5 /uL
Albumin, CSF	295	0 - 35 mg/dL
Glucose, CSF	59	40 - 70 mg/dL
Oligoclonal Bands, CSF	0	0 - 1 Bands
Oligoclonal Bands, Serum	0	0 - 1 Bands
Immunoglobulin G, CSF	49.3	0 - 6.0 mg/dL
Myelin Basic Protein, CSF	1.50	0.00 - 5.50 ng/mL
Myelin Oligodendrocyte Glycoprotein (MOG) IgG Screen	<1:10	<1:10
Neuromyelitis Optica / AQP4 IgG	<1:10	<1:10

**Table 6:** CSF microbiology panel K1, strain of E Coli; PCR, polymerase chain reaction; Strep, streptococcus; CMV, cytomegalovirus; HSV 1, herpes simplex virus type 1; HSV 2, herpes simplex virus type 2; HHV6, human herpesvirus 6; VDRL, venereal disease research laboratory test; Ag, antigen

COMPONENT	VALUE	REFERENCE RANGE
E Coli K1 PCR	Not Detected	Not Detected
Influenzae PCR	Not Detected	Not Detected
Listeria Monocytogenes PCR	Not Detected	Not Detected
Neisseria Meningitidis PCR	Not Detected	Not Detected
Strep B CSF	Not Detected	Not Detected
Strep Pneumoniae PCR	Not Detected	Not Detected
CMV PCR	Not Detected	Not Detected
Enterovirus PCR	Not Detected	Not Detected
HSV 1 PCR	Not Detected	Not Detected
HSV 2 PCR	Not Detected	Not Detected
HHV6 PCR	Not Detected	Not Detected
Parechovirus PCR	Not Detected	Not Detected
Varicella Zoster PCR	Not Detected	Not Detected
Cryptococcus PCR	Not Detected	Not Detected
VDRL	Non-Reactive	Non-Reactive
Cryptococcal Ag	Negative	Negative
Amoeba	No Organisms Observed	No Organisms Observed

Per the Neurology report, given that the patient was in the postpartum state, she was unable to receive IVIG due to the risk of thrombosis. Thus, her care plan consisted of five daily plasmapheresis sessions. The patient was prepared for surgery, and a 15 cm trialysis catheter was placed in the right internal jugular vein using the modified Seldinger technique.

On postpartum day 5, the patient received the first session of plasmapheresis and tolerated it well. She continued with her remaining four sessions on postpartum days 6, 7, 8, and 9, respectively. During these days, the patient care team reported her condition improved slightly, but the weakness and sensory deficits remained. On postpartum day 9, the patient finished her trials of plasmapheresis. Due to her continued deficits, hematology ordered two more sessions of plasmapheresis for postpartum days 10 and 11. She was referred for transfer to an inpatient physical medicine and rehabilitation program; however, this was denied by her insurance. 

Discharge planning on postpartum day 11 was discussed with the patient, and it was decided that she would continue to receive physical therapy in an outpatient setting two to three times a week until her symptoms improved. It was also explained to the patient that there are two types of GBS: monophasic form and chronic relapsing. If she were to relapse within the next month post discharge, her future care would involve monthly immunotherapy with intravenous immunoglobulin (IVIG) for the chronic relapsing form of GBS.

## Discussion

GBS is extremely rare in pregnancy, with only about 1.2-1.9 cases per 100,000 people reported annually. Although it can occur at any time during pregnancy, it occurs most often in the third trimester and first two weeks postpartum [3]. Current literature suggests that GBS does not affect fetal development or increase the risk of spontaneous abortion. However, worsening neurological symptoms may prompt the need for immediate delivery. One case report details a 34-year-old female at 33 weeks’ gestation who was misdiagnosed with an acute viral respiratory infection and nasopharyngitis after presenting with headache, nausea, vertigo, paresthesia, weakness, and muscle pain [6]. She was later diagnosed with GBS after experiencing trouble swallowing, numbness of the tongue, and paresthesia of the anterior abdominal wall. Despite treatment, the bulbar syndrome and other neurologic symptoms worsened, prompting the need for an emergency cesarean section.

Furthermore, GBS increases the risk of maternal neuromuscular respiratory failure, with as many as 34.5% of pregnant women with GBS needing ventilator support [3]. Another case report details the diagnosis of GBS in the 31st week of pregnancy after presenting with a recent diarrheal infection, bulbar syndrome, neck muscle weakness, and paresthesia of the bilateral upper extremities [7]. The patient required intubation for respiratory failure due to rapid worsening of symptoms. Patients with clinical signs of respiratory failure such as shortness of breath, tachypnea, abnormal arterial blood gases, and use of accessory muscles should be closely monitored and admitted to an intensive care unit (ICU).

While the exact pathogenesis of GBS is not fully understood, it is thought to be caused by molecular mimicry between gangliosides in Schwann cells and epitopes found in the cell walls of some microorganisms [8]. Given that it commonly presents with non-specific symptoms such as pain, weakness, numbness, and paresthesia, proper diagnosis of GBS in pregnancy is often delayed, as those symptoms can overlap with common pregnancy-related changes/symptoms. Common differential diagnoses of GBS include sciatica, carpal tunnel syndrome, pregnancy-related changes, transverse myelitis, myasthenia gravis, botulism, and Lyme disease. To reduce the morbidity and mortality associated with GBS, the Brighton criteria was created for prompt diagnosis and treatment of the disease. It describes the absence of an alternate diagnosis for weakness, hypo- or areflexia, monophasic course, bilateral and flaccid weakness of limbs, normal CSF cell count, high CSF protein levels, and nerve conduction studies suggestive of GBS (Table 7) [9]. The presence of elevated CSF protein with a normal white blood cell count is known as albuminocytologic dissociation.

**Table 7:** Brighton criteria for GBS +, presnt; -, absent; +/-, present or absent; CSF, cerebrospinal fluid; GBS, Guillain Barré Syndrome; NCS, nerve conduction study Table Adapted from: Ghazanfar et al., 2020 [9]; Creative Commons Attribution License CC-BY 4.0

Diagnostic Criteria	Level of Diagnostic Certainty			
	Level 1	Level 2	Level 3	Level 4
Absence of alternative diagnosis for weakness	+	+	+	+
Diminished or absent deep tendon reflex in weak limbs	+	+	+	+/-
Monophasic course and time between onset and nadir, 12 hours to 28 days	+	+	+	+/-
Bilateral and flaccid weakness of limbs	+	+	+	+/-
CSF cell count < 50 cells/microL	+	+	-	+/-
CSF protein concentration > normal value	+	+/-	-	+/-
NCS findings consistent with one of the subtypes of GBS	+	+/-	-	+/-

Treatment of GBS in patients who are not ambulating is immunotherapy via either IVIG or plasma exchange (PLEX). Current literature suggests that both IVIG and PLEX are safe and effective treatments for improving muscle strength and reducing the need for mechanical ventilation in patients with severe GBS [10]. Functional recovery after GBS is a gradual process, with 80% of patients regaining independent ambulation and more than half recovering completely within one year [11]. The risk of being unable to walk at six months can be estimated using the Erasmus GBS outcome score (EGOS) system, which considers age, preceding diarrhea, and a GBS disability score to predict patient outcomes [12]. However, this system was created from data compiled from a White Dutch population and may not apply to all patients.

## Conclusions

This case highlights the presence of two medical conditions which could both explain the weakness, numbness, and tingling experienced by the patient. Pregnancy brings about a myriad of physiologic changes that can alter the center of gravity, sensation, and muscle strength. However, the compilation of severe, bilateral muscle weakness, paresthesia, areflexia, and recurrent falls in our patient suggested a more intense picture beyond just the woes of pregnancy. Thus, a detailed workup including a lumbar puncture, several MRIs, and neurology evaluation was required. The patient was then treated with seven rounds of plasmapheresis to treat her disease. However, even after an 11-day hospital course, the patient will need to be followed with outpatient physical therapy and monitoring for relapse. Although pregnancy could have accounted for some of the patient’s symptoms, the severity and duration required further evaluation, which led to the correct diagnosis of GBS during pregnancy. This case demonstrates the need to educate both obstetricians and emergency room physicians on the varying presentations of GBS, so it can remain high on the list of differential diagnoses in the setting of unexplained weakness, numbness, and tingling.
